# An ALG12-CDG patient with a novel homozygous intronic mutation associated with low ALG12 mRNA

**DOI:** 10.1186/s13023-025-03535-4

**Published:** 2025-02-21

**Authors:** Sandrine Vuillaumier-Barrot, Thierry Dupré, Tiffany Andriantsihoarana, Vincent Desportes, David Cheillan, Stuart E. H. Moore, Isabelle Chantret

**Affiliations:** 1 Université Paris Cité, INSERM U1149, 16 Rue Henri Huchard, 75018 Paris, France; 2 Département de Biochimie Et de Génétique, AP-HP, Hôpital Bichat, 46 Rue Henri Huchard, 75018 Paris, France; 3 Service de Neuropédiatrie, Hospices Civils de Lyon, 59 Boulevard Pinel, 69677 Bron Cedex, France

**Keywords:** Congenital Disorders of Glycosylation, N-Glycosylation, Dolichol-linked-oligosaccharide, RFT1, ALG12, Intronic variant

## Abstract

**Background:**

Type I Congenital Disorders of Glycosylation (CDG-I) are inherited diseases presenting deficits in protein *N*-glycosylation involving either the biosynthesis of the lipid-linked oligosaccharide Glc_3_Man_9_Glc*N*Ac_2_-PP-dolichol or transfer of its oligosaccharide to protein.

**Results:**

We describe a patient harbouring hypoglycosylated transferrin, a characteristic of CDG-I. NGS revealed a homozygous *RFT1* (c.16G > T p.Val6Leu) variant of unknown significance that is predicted to be benign. Metabolic radiolabelling of the patient’s fibroblasts did not reveal the accumulation of truncated Man_5_Glc*N*Ac_2_-PP-dolichol expected of RFT1-CDG but rather an accumulation of Man_7_Glc*N*Ac_2_-PP-dolichol, characteristic of ALG12-CDG. Revaluation of the NGS data revealed a homozygous (22_50311909A_G, c.-79 + 2 T > C) variant that modifies the second nucleotide of the first intron of the *ALG12* gene upstream of the first coding exon (exon 2). Sequencing of *ALG12* cDNA revealed a 4-base insertion between exon 1 and exon 2 suggesting a shift in mRNA splicing in this intron to a putative new GU donor site. The patient’s fibroblasts display 3% of control *ALG12* mRNA levels.

**Conclusion:**

This is the first description of a pathogenic intronic *ALG12* variant upstream of the first coding exon. The modification of the splicing process between intron 1 and exon 2, the very low transcript level and the absence of other mutations in the patient's ALG12 gene lead us to conclude that this *ALG12* variant is a predicted Loss of Function (pLOF) variant.

## Background

Congenital Disorders of Glycosylation (CDG) are a group of inborn errors of metabolism affecting glycoconjugate biosynthesis (1). Historically, the first CDG descriptions concerned mutations of the protein N-glycosylation pathway. Based upon SDS-PAGE / isoelectric focusing patterns of hypoglycosylated serum glycoproteins and the subcellular location of the defective enzyme, two types of CDG were delineated: type I CDG (CDG-I) with errors in the synthesis and transfer of the dolichol linked oligosaccharide (DLO) onto the peptide chain in the endoplasmic reticulum (ER), and type II CDG (CDG-II) involving altered N-glycan maturation in the Golgi apparatus (2). To date, 29 CDG-I and 21 CDG-II subtypes have been described, and named according to the defective gene. Recent nomenclature (*GENE*-CDG) does not make the distinction between CDG-I and -II.

The clinical presentations of CDG overlap between subtypes but can differ between patients with the same mutation. Signs may include multi organ dysfunction as well as cerebellar malformations of variable severity (3). The neurological involvement affects the central and peripheral nervous system: generalized hypotonia, strabismus, cerebellar atrophy or progressive cerebellar hypoplasia, moderate to severe mental retardation, retinitis pigmentosa, peripheral neuropathy, and frequent seizures (4).

In the present study, we describe a patient presenting signs of CDG syndrome and a pattern of hypoglycosylated serum glycoproteins consistent with a biochemical defect in an early ER-situated step of the protein N-glycosylation pathway. NGS revealed new homozygous variations of unknown significance in genes required for DLO biosynthesis: one in the *RFT1* gene (c.16G > T p.Val6Leu rs748893039 NM_052859.4) and the other in the *ALG12* gene (22_50311909A_G, c.−79 + 2 T > C NM_024105.4). In silico predictions, functional studies, mRNA quantitation and cDNA sequencing indicate the *RFT1* variant to be benign whereas the *ALG12* variant not found in gnomAD, associated with a severe reduction in *ALG12* mRNA expression, is shown as a loss-of-function variant and classified as pathogenic class 4 according to ACMG guidelines.

## Materials and methods

### Materials

Cytiva™ Ficoll-Paque™ PLUS Media and the Verso cDNA synthesis kit protocol were obtained from Thermo Fisher ScientificTM (Waltham, USA). INTEGRAGEN with NEBNext Ultra II DNA Library Prep Kit for Illumina was from New England BioLabs (Evry, FR). C18 SepPak cartridges was purchased from Waters (Guyancourt, FR). D-[2-^3^H]mannose (20 Ci/mmole) was obtained from Biotrends (Cologne, DE). En3hance spray and the kit chemagic STAR DNA Blood4k were purchased from PerkinElmer Life Sciences (Zaventem, BE). AG-1/AG50 columns, X-OMAT AR film and PNGase F from *Elizabethkingia meningoseptica* were obtained from SIGMA–Aldrich SARL (St Quentin Fallavier, FR). Thin Layer Chromatography (TLC) plates were purchased from MERCK (Darmstadt, DE). RNeasyⓇ Plus Mini Kit (50) was obtained from Qiagen (Courtaboeuf, FR). LightCyclerⓇ 480 SYBRⓇ Green I Master was purchased from Roche TM (Mannheim, DE).

### Leukocyte isolation

Leukocytes were isolated from blood using a Ficoll-Paque Plus gradient. The genomic DNA was extracted from leukocytes employing the kit chemagic STAR DNA Blood4k according to the manufacturer’s instructions.

### Cell culture

Skin biopsy fibroblasts from control and the patient, were cultured in RPMI medium supplemented with 10% FCS and 1% penicillin/streptomycin in 75 cm^2^ flasks, at 37 °C in a humid atmosphere containing 5% CO_2_.

#### Western-blot of serum glycoproteins and enzyme activities

Western blotting of 4 different human serum glycoproteins (orosomucoid, alpha-antitrypsin, transferrin, and haptoglobin) were performed as previously described (5). Phosphomannomutase (6,7) and phosphomannose isomerase (8) activities were performed on leukocytes as previously described.

### Capillary Zone Electrophoresis

Transferrin glycoforms were analyzed by capillary zone electrophoresis using the Capillarys Capiflex 2 automated system manufactured by Sebia (Lisses, France).

### Mutation analysis

Next-generation sequencing (NGS) of 42 genes involved in CDG, was performed. Whole genome sequencing was performed by INTEGRAGEN with NEBNext Ultra II DNA Library Prep Kit according supplier recommendations, on an Illumina NovaSeq as Paired End 150 reads. The pathogenicity of the missense variant was evaluated with ACMG criteria: gnomAD frequency, in silico prediction and recommendations from the ClinGen SVI Splicing subgroup for interpretation of loss-of-function (LOF) variants. Functional data was necessary to prove a LOF *ALG12* pathogenic variant.

### Metabolic radiolabeling of cells

Fibroblasts established from skin biopsy from a control subject and from the patient, were pulse-radiolabelled as previously described (9). Briefly, cells layers were washed twice with PBS and once with labelling medium consisting of glucose-free RPMI 1640 supplemented with 1 mM Glc, 2% dialysed FCS and 2 mM fucose. The cells were then incubated for 30 min at 37 °C in 1 mL of labelling medium containing 100 µCi of [2-^3^H]mannose (20 Ci / mmol). The reaction was stopped by adding 12 mL PBS (4 °C).

### Extraction of Dolichol-linked oligosaccharides (DLO)

DLO were isolated by the Folch extraction method as in (9). After removing the last PBS wash, cells were scrapped in the presence of 2 mL of extraction buffer containing 100 mM Tris HCl / 4 mM MgCl_2_ (pH 7.4) and methanol (1:2, v/v). The 2 ml cell extracts were collected in 15 ml tube on which the same volume of chloroform (CHCl_3_) was added. The tubes were then shaken vigorously, and after standing for 30 min at room temperature and centrifugation (room temperature, 1150 g, 5 min), the upper methanolic phase, the lower CHCl_3_ phase and the proteins at the interface, were recovered. After washing with methanol, 3 extractions of the protein interface were performed by adding a CHCl_3_/methanol/H_2_O mixture (10:10:3, v/v/v). Three 10/10/3 extractions were combined with the previously isolated CHCl_3_ phase to give a fraction containing total DLO.

### Recovery of oligosaccharides from both radioactive DLO and glycoproteins

The CHCl_3_/10:10:3 samples were dried under vacuum and lipids were solubilized with 200 µL tetrahydrofuran. 2 mL of 22 mM HCl was added to the tubes and incubated at 100 °C for 45 min in order to release the oligosaccharides from the DLO. The glycoproteins from the 10:10:3 extracted protein pellet were submitted to Peptide N-glycanase digestion as described in the manufacturer’s instructions. Released N-glycans were desalted on AG-1/AG50 columns and further purified by passage through C18 SepPak cartridges. After drying under vacuum oligosaccharides were resolved by thin-layer chromatography (TLC) on silica-coated plastic sheets (0.2 mm thickness) that were developed in *n*-propanol/acetic acid/water, 3/3/2 for 20 h. Radioactive components were detected on X-OMAT AR film by fluorography after spraying the TLC with En^3^hance.

### RT-qPCR ALG12

RNAs were extracted from control and patient fibroblasts according to the protocol from the RNeasyⓇ Plus Mini Kit (50) (QiagenTM). Retro-transcription (RT) was performed according to the Verso cDNA synthesis kit protocol (Thermo scientificTM) using 1 µg of RNA.

The cDNA sequence of *ALG12* from exon 1 to exon 10 was amplified from 1 or 2 µl of the RT reaction by classic PCR using the primers F1 5’-TCGGGCTCTGTTCTGTTTCC-3’ and R1 5’-CCCTCGTTCTTTGGTGCTGA-3’ and further sequenced on both strands. Two couples of ALG12 primers were used for RT-QPCR: F2 5’-GCCAGTGGTGATCGCAGT-3’/R2 5’-CGAGTCCAAGCACTCCTCTAA-3’ and F3 5’-CCCATGCTCAACATCACG-3’/R3 5’- GCTTTGTACAGCCAAGACTTTTT-3’ for getting an amplicon partially covering respectively exon 3/exon 4 and exon 7/exon 8. QPCR was performed with the LightCyclerⓇ 480 SYBR Green I Master (RocheTM). In order to normalize the QPCR, the cDNA of the housekeeping gene ꞵ2M (ꞵ−2-microglobulin) was amplified with the F8 5’-GAGCATTCAGACTTGTCTTTCAGC-3’ / R8 5’-AATTCATCCAATCCAAATGCG-3’ primer couple. The *ALG5* cDNA was amplified as an internal control with the F7 5’-TACACGTTGAACGATGGGCA-3’/R7 5’-TTCCGAGTTTGCTCAAGCCT-3’ primer couple.

## Results

### Clinical presentation

The patient is a girl born of consanguineous parents who have three other children in good health. The pregnancy was unremarkable except that metrorrhagia was noted at 36 weeks, and an emergency caesarean section was performed. Birth weight was 2400 g, birth length 46 cm and head circumference 32 cm. Apgar score was 10 / 10 at 1, 5 and 10 min after birth. Good neonatal adaptation was observed. Despite psychomotor delay (head posture after 6 months, sitting posture at 1 year, walking at 27 months) a cerebral MRI scan, carried out at the age of two years, was normal without hypoplasia of the cerebellum. The last clinical examination was made at 6 years and 3 months of age, and at that time, she had no expressive language and presented with severe intellectual disability. She walked with the widening of the sustentation polygon and hyperlordosis, with valgus deformity of the feet in the absence of pyramidal signs. The head circumference was at −3 sd, weight was 18.4 kg (−0.5DS) and height was 109.5 cm (−1DS). She presented with convergent strabismus and bilateral hyperopia at + 3.75 diopters.

### Clinical biochemistry data

**Fig. 1:**
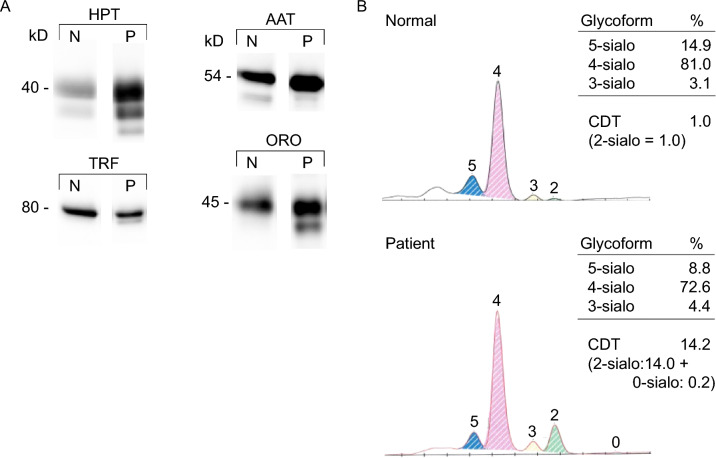
**A** Analysis of serum Glycoproteins. Plasma samples from normal subject (N), and the patient (P) were submitted to SDS PAGE followed by Western-blot analysis of 4 seric glycoproteins: Haptoglobin (HPT), Orosomucoid (ORO), alpha-antitrypsin (AAT) and transferrin (TRF). The molecular weights are indicated to the left of the gel. **B** Capillary zone electrophoresis profiles (CZE) of transferrin obtained from normal (N) and patient (P) subjects. The percentage of 5-sialo, 4-sialo, 3-sialo, 2-sialo, 0-sialo corresponding to penta-, tetra-, tri-, di- and asialotransferrin, respectively, are indicated on the right side of each CZE profiles

Congenital galactosemia was ruled out and no coagulation abnormality was observed. Western Blot of 4 serum glycoproteins (orosomucoid, alpha-antitrypsin, transferrin, and haptoglobin) revealed hypoglycosylated isoforms (Fig. 1A). The transferrin electrophoretogram from a healthy subject shows a major tetrasialylated form (N: 78–86%) and also minor 5-sialo (N: 11–18%), 3-sialo (N < 6%) and −2-sialo (N < 1.3%) forms. The 1- sialo and 0-sialo forms are not detectable (Fig. 1B). By contrast the patient’s transferrin electrophoretogram reveals that the 2-sialo form (14%) is above the normal range, and an appearance of 0-sialo (0.2%) (Fig. 1B). These data indicate the absence of entire disialylated glycans rather than reduced sialylation of glycans, suggesting an early ER-situated step of the protein N-glycosylation pathway is at fault. Phosphomannomutase and phosphomannose isomerase deficiencies are among the most common causes of impaired DLO synthesis in the ER, but were normal in leukocytes from the patient.

#### An RFT1 variant of unknown significance

**Fig. 2:**
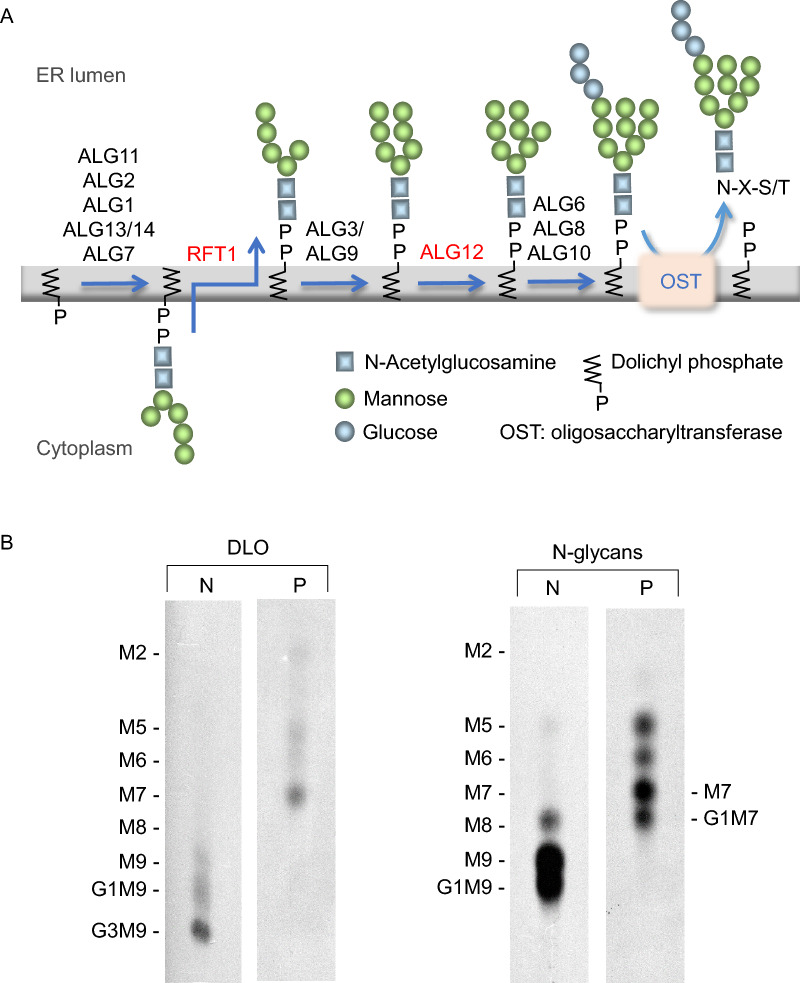
**A** Schematic presentation of DLO synthesis in the RE. An accumulation of DLO containing GlcNAc_2_Man_5_ or GlcNAc_2_Man_7_ will be expected for a RFT1-CDG patient and an ALG12-CDG patient respectively. OST: oligosaccharide transferase. **B** Analysis of DLO biosynthesis and *N*-glycans transfer. After pulse radio-labelling with 2-[^3^H-mannose], the oligosaccharides linked to dolichol or glycoproteins were purified as described in material and methods and further resolved by TLC. The abbreviations used on the left side are: *M2*, Man_2_GlcNAc_2_; *M5*, Man_5_GlcNAc_2_; *M6*, Man_6_GlcNAc_2;_ *M7*_,_ Man_7_GlcNAc_2;_ *M8,* Man_8_GlcNAc_2;_ *M9*, Man_9_GlcNAc_2_; *G1M9*, GlcMan_9_GlcNAc_2;_ *G3M9*, Glc_3_Man_9_GlcNAc_2_

Targeted next-generation sequencing (NGS) of 42 genes involved in CDG was performed. A previously unreported *RFT1* variant (c.16G > T p.Val6Leu) of unknown significance was detected and predicted to be benign by SIFT and Polyphen2. The *RFT1* gene encodes a protein of unknown function that is involved in the elongation of Man_5_GlcNAc_2_-PP-dolichol (Fig. 2A). Metabolic radiolabelling of cells from RFT1-CDG patients with [2-^3^H]mannose reveals the accumulation of [2-^3^H]Man_5_GlcNAc_2_-PP-dolichol. Some of these truncated glycolipids that escape the blockade are elongated normally to [2-^3^H]Glc_3_Man_9_GlcNAc_2_ and transferred to protein, leading to a normal N-glycan profile (10–12). Using this approach here, it can be seen that control fibroblasts yield predominantly the mature DLO, [2-^3^H]Glc_3_Man_9_GlcNAc_2_-PP-dolichol, as expected, whereas fibroblasts from the patient reveal predominantly [2-^3^H]Man_7_GlcNAc_2_-PP-dolichol and lesser amounts of intermediates containing 2 – 6 residues of mannose (Fig. 2B). Normally, after transfer to protein the [2-^3^H]Glc_3_Man_9_GlcNAc_2_ N-glycan is rapidly processed to yield [2-^3^H]Glc_1-0_Man_9-8_GlcNAc_2_ structures, and these are seen on glycoproteins derived from control cells. By contrast, glycoproteins from patient cells reveal predominantly [2-^3^H]Glc_1-0_Man_7_GlcNAc_2_ N-glycans with smaller amounts of N-glycans comigrating with oligosaccharides containing 5 – 6 residues of mannose (Fig. 2B). Therefore, these data point to a deficiency of dolichyl-P-Man: Man_7_GlcNAc_2_-PP-dolichol alpha-6 mannosyltransferase encoded by the *ALG12* gene (Fig. 2B) (9,13–16).

#### Identification of a novel ALG12 intronic variant

**Fig. 3:**
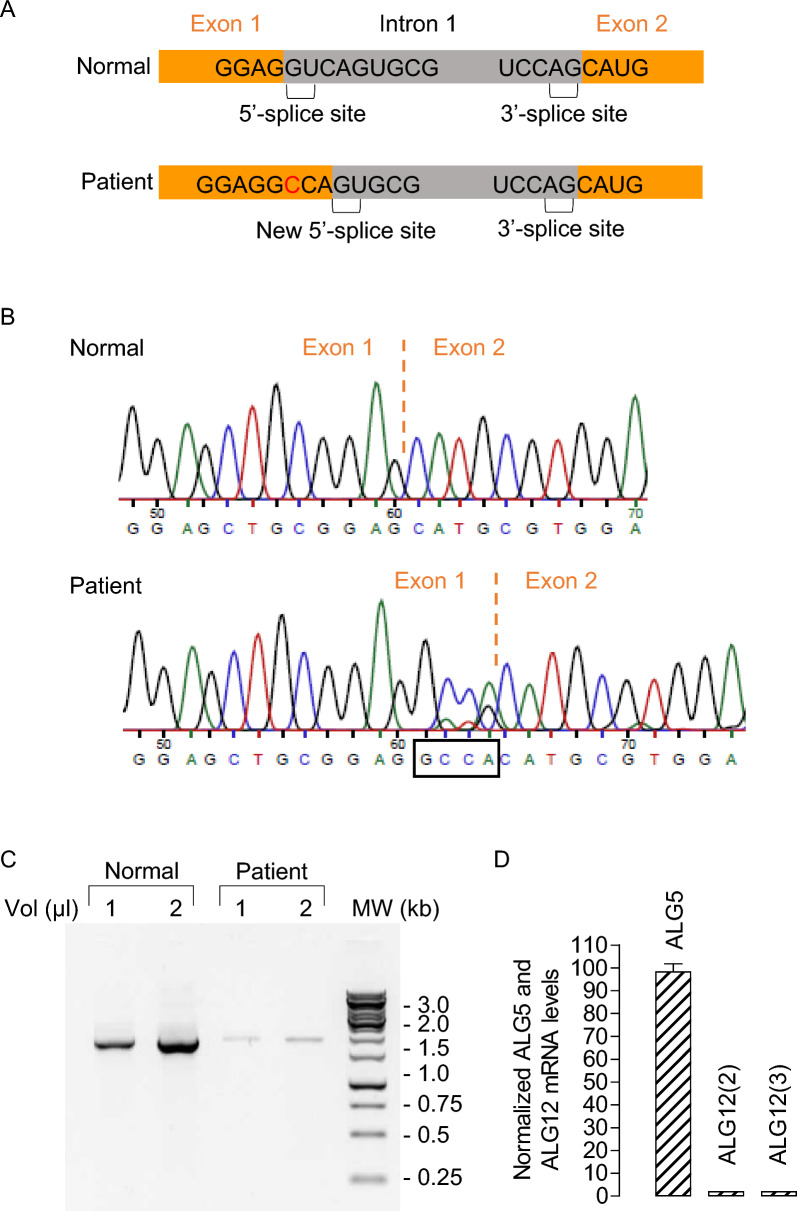
**A** Scheme of ALG12’s intron 1 from Normal (N) and Patient (P) subjects. Mutation of the second nucleotide of the normal splice donor sequence between exons 1 and 2 will unmask a new putative splice donor and will result in a 4 base insertion in the cDNA (see below). **B**. *ALG12* cDNA sequence at the exon 1/exon 2 junction from Normal (N) and Patient (P) subjects. Note the 4 base insertion at the end of intron 1 in the patient. **C** *ALG12* PCR products generated from Normal (N) and Patient (P) cDNA. PCR amplification from 1 or 2 µl of cDNA templates was performed as described in materials and methods. The use of primers S1/R1 allows the amplification of the whole coding sequence of *ALG12* mRNA and 2 kb PCR products were loaded on a 1% agarose gel. The molecular weight (MW) are indicated to the right side of the gel. **D** Analysis of ALG12 and ALG5 expression in the patient. *ALG12* and *ALG5* mRNA have been amplified by RT-QPCR from cDNA templates from control, and patient fibroblasts as described in material and methods. Note that 2 different couples of primers have been used for the amplification of *ALG12* cDNA: for ALG12 (2), the S2/R2 pair of primers was used which allows an amplification straddling exon 3 and exon 4, meanwhile for ALG12 (3), the S3/R3 primers were used and allow amplification between exon 7 and exon 8

Upon re-examination of the NGS data, an *ALG12* intronic variant (NM_024105.4 (*ALG12*):c.−79 + 2 T > C), previously unreported in gnomAD, affecting the donor site of intron 1 was noted (see Fig. 3A). Whole genome sequencing revealed no additional pathogenic data. Sequencing of the *ALG12* cDNA from the patient shows an insertion of four additional bases between exon 1 and exon 2 (Fig. 3B). This insertion should not change the open reading frame, since it takes place 79 bp upstream of the ATG codon, but could modify the 5’-splice site (donor site) between exon 1 and exon 2. The additional 4 nucleotides found in the patient's cDNA (GCCA) sequence are identical to the first 4 nucleotides of intron 1 (Fig. 3A and B). The following GT in intron 1 could then constitute a potential new 5'-splice site (Fig. 3A). Recommandations from the ClinGen SVI Splicing Subgroup framework for interpretation of LOF variants would suggest that this variant is frame-preserving (17). However, conventional RT-PCR of *ALG12* cDNA from exon 1 to 10 showed a very low amplification in the patient compared to the control (Fig. 3C). Furthermore, RT-QPCR with 2 couples of primers showed a 33-fold decrease of *ALG12* mRNA expression when compared to that of control fibroblasts, while *ALG5* expression appeared to be normal (Fig. 3D). Thus, regardless of the position of the primers, the level of the *ALG12* transcript in fibroblasts from the patient is particularly reduced compared to that of the control cells.

## Conclusion

This patient has the biochemical characteristics of an ALG12-CDG patient, and carries the first described intronic variant which is upstream of the first coding exon. This mutation is associated with a 33-fold reduction in *ALG12* mRNA level, which could be the consequence of a recently described splicing-coupled mRNA quality control process (18,19). The modification of the splicing process between intron 1 and exon 2, the very low transcript level and the absence of other mutations in the patient's ALG12 gene lead us to conclude that this variant is a new predicted Loss of Function (pLOF) variant classified as pathogenic class 4 according to ACMG guidelines.

## Data Availability

All biochemistry data generated or analysed during this study are included in this published article. The sequencing data sets are available from the corresponding author on reasonable request.

## Abbreviations

CDG: Congenital Disorders of Glycosylation
cDNA: Complementary DNA
DLO: Dolichol Linked Oligosaccharide
DNA: Desoxyribonucleique Acid
ER: Endoplasmic Reticulum
FCS: Fetal Calf Serum
Glc: Glucose
Leu: Leucine
Man: Mannose
MRI: Magnetic Resonance Imaging
mRNA: Messenger RNA
NGS: Next Generation Sequencing
ORF: Open Reading Frame
pLOF: Predicted Loss of Function
PNGase: Peptide N-Glycanase
PCR: Polymerase Chain Reaction
QPCR: Quantitative PCR
RNA: Ribonucleique Acid
RPMI medium: Roswell Park Memorial Institute medium
RT: Reverse transcriptase
TLC: Thin Layer Chromatography
Val: Valine
